# The impact of postoperative adjuvant therapy on EGFR-mutated stage IA lung adenocarcinoma with micropapillary pathological subtypes

**DOI:** 10.1186/s12957-024-03429-y

**Published:** 2024-09-05

**Authors:** Ran Cheng, Zhexue Hao, Li Qiu, Xiang Zheng, Sihe Huang, Jianzhao Xian, Haoyang Huang, Jianfu Li, Zhenhui Zhang, Kaiwen Ye, Wentao Wu, Yaowen Zhang, Jun Liu

**Affiliations:** 1 Department of Thoracic Surgery and Oncology, China State Key Laboratory of Respiratory Disease & National Clinical Research Center for Respiratory Disease, the First Affiliated Hospital of Guangzhou Medical University, Guangzhou, China; 2 Department of Oncology, The First Clinical Medical College of Henan University, Kaifeng, China; 3 Department of Radiation Oncology, Anyang Tumor Hospital, The Affiliated Anyang Tumor Hospital of Henan, Henan Medical Key Laboratory of Precise Prevention and Treatment of Esophageal Cancer, University of Science and Technology, Anyang, China

**Keywords:** Lung adenocarcinoma, Micropapillary, EGFR, Adjuvant therapy, Prognosis

## Abstract

**Background:**

Micropapillary (MPP) adenocarcinoma is considered one of the most aggressive pathological types of lung adenocarcinoma (LADC). This retrospective study aimed to evaluate the prognostic significance and benefit of postoperative adjuvant therapy (PAT) in stage IA LADC patients with different proportions of MPP components.

**Materials and methods:**

We retrospectively examined clinical stage IA LADC patients who underwent surgical resection between August 2012 and December 2019. In terms of the proportion of MPP components (TPM), the tumors were reclassified into three categories: MPP patterns absent (TPMN); low proportions of MPP components (TPML); and high proportions of MPP components (TPMH). The dates of recurrence and metastasis were identified based on physical examinations and were confirmed by histopathological examination.

**Results:**

Overall, 505 (TPMN, *n* = 375; TPML, *n* = 92; TPMH, *n* = 38) patients harboring EGFR mutations were enrolled in the study. Male sex (*P* = 0.044), high pathological stage (*P* < 0.001), and MPP pathological subtype (*P* < 0.001) were more frequent in the TPM-positive (TPMP) group than in the TPM-negative (TPMN) group. Five-year disease-free survival (DFS) rates were significantly lower in the TPMP group than in the TPMN group (84.5% vs. 93.4%, *P* = 0.006). In addition, patients with high proportions (greater than 10%) of MPP components had worse overall survival (OS) (91.0% vs. 98.9%, *P* = 0.025) than those with low proportions (5%≤ TPM ≤ 10%). However, postoperative EGFR tyrosine kinase inhibitors (TKIs) or adjuvant chemotherapy (ACT) cannot improve DFS and OS between EGFR-mutated patients with different proportions of MPP components.

**Conclusion:**

MPP was related to earlier recurrence and shortened survival time, even in stage IA. Further research needs a larger sample size to clarify that EGFR-mutated stage IA patients with MPP components obtain survival benefits from adjuvant therapy.

**Supplementary Information:**

The online version contains supplementary material available at 10.1186/s12957-024-03429-y.

## Background

For patients with early-stage non-small cell lung cancer (NSCLC), surgery remains the most promising treatment [1]. However, even after complete surgical resection, there is still a risk of cancer recurrence and distant metastasis [2, 3]. Recent findings from the ADAURA trial have shown that epidermal growth factor receptor tyrosine kinase inhibitors (EGFR-TKIs) significantly improve DFS duration by approximately 18 months in stage IB lung adenocarcinoma (LADC) patients with EGFR mutations [4]. However, the use of adjuvant therapy after surgery for EGFR-mutated patients with stage IA NSCLC is still not supported by sufficient evidence.

Many studies have shown the effect of the presence of a micropapillary (MPP) subtype on a poorer prognosis, even in stage I LADC patients [5–8]. Patients with a significant MPP component (equal to or greater than 5%) in their surgical specimen are considered at high risk for cancer recurrence and metastasis [9]. While adjuvant therapy is not routinely recommended for stage IA NSCLC patients in current clinical practice [10], more aggressive treatment approaches are needed to control disease progression in MPP-predominant cases.

In this study, we aimed to explore the clinicopathological characteristics and survival outcomes of EGFR-mutated stage IA LADC patients with different proportions of MPP components. Specifically, we investigated whether these high-risk patients in the early stage can benefit from EGFR-TKIs or adjuvant chemotherapy (ACT). By understanding the potential benefits of postoperative adjuvant treatment (PAT) in this specific subgroup, we hope to provide insights that can improve their overall prognosis and guide treatment decisions.

## Materials and methods

### Study design and population

From August 2012 to December 2019, we retrospectively reviewed all patients who underwent complete resection and histologically confirmed stage IA LADC at the First Affiliated Hospital of Guangzhou Medical University, and all EGFR-mutant patients were included in this study. The exclusion criteria of this study were as follows: (1) patients with multiple primary carcinomas; (2) patients whose tumor tissue for immunohistochemistry and genetic testing was insufficient; (3) patients with incomplete clinical data or follow-up information; (4) EGFR wild-type patients.

### Data collection

Data on patient demographics were collected from the medical records, including age, sex and smoking history. Occasional gaps in the interview records led to certain cases being classified as unknown for smoking history. Cancer information was documented in pathological reports and included tumor laterality, resection type, T stage and EGFR mutation status. The type of surgical resection was categorized into lobectomy or sublobectomy, which included segmentectomy and wedge resection. Tumor staging was classified according to the eighth edition of the TNM classification of the International Association for the Study of Lung Cancer. Pathological types and EGFR mutation status were derived from surgical specimens. EGFR mutations were identified by either next generation sequencing or the polymerase chain reaction (PCR) method.

Postoperative follow-up chest CT scans were performed every 3 to 6 months for the first two years and annually thereafter until progressive disease or death. The dates of recurrence and metastasis were identified based on physical examinations and were confirmed by histopathological examination. The primary endpoint of this study was the 5-year DFS, defined as the duration from the initiation of operation to the first recurrence or death. The secondary endpoint was overall survival (OS), defined as the duration from the initiation of operation to death from any cause. To gather data on patients without recorded survival status in the postoperative medical records, we conducted telephone interviews to assess their overall well-being and disease status.

### Histologic evaluation

All specimens were routinely fixed in formalin and stained with hematoxylin and eosin (HE). Two pathologists together reviewed an average of 8 (range 4–12) slides per patient using a multiheaded microscope. Tumors were classified into 5 distinctive subtypes based on the International Association for the Study of Lung Cancer (IASLC)/American Thoracic Society (ATS)/European Respiratory Society (ERS) classification criteria as (1) acinar, (2) lepidic, (3) solid, (4) micropapillary (MPP), (5) papillary. The ratio of each histological component was calculated in 5% increments. The largest proportion of a combination of histological patterns was identified as the predominant subtype, and the lowest limit for the predominant subtype was set at 30%.

In terms of the proportion of MPP components (TPM), patients were divided into two groups: TPM-negative (TPMN, tumors without MPP subtype) and TPM-positive (TPMP, tumors with MPP subtype). Then, in the defined positive group, tumors with 5% ≤ TPM ≤ 10% were classified as TPM-low (TPML), and those with TPM greater than 10% were classified as TPM-high (TPMH). Lung cancer staging was performed for all patients according to the eighth tumor node metastasis (TNM) staging classification.

### Statistical analysis

The χ2 test was used to compare the characteristics of patients for categorical variables. The Pearson test was used to determine the correlation. DFS and OS curves were plotted by the Kaplan-Meier method, and the log-rank test was used to evaluate the differences among the subgroups. Univariate and multivariate analyses were used to assess the effect of the covariates on DFS and OS. The HR and 95% CI were estimated using the Cox proportional hazards model. The variables with *P* < 0.05 on univariate analysis were used as the input variables for the multivariate analysis. All statistical analyses were carried out using SPSS software, version 23.0 (IBM Corporation, Armonk, NY, USA) and R version 4.3.1 (R Development Core Team, Vienna, Austria). Statistical significance was considered as *P* values less than 0.05.

## Results

### Patient characteristics

**Fig. 1:**
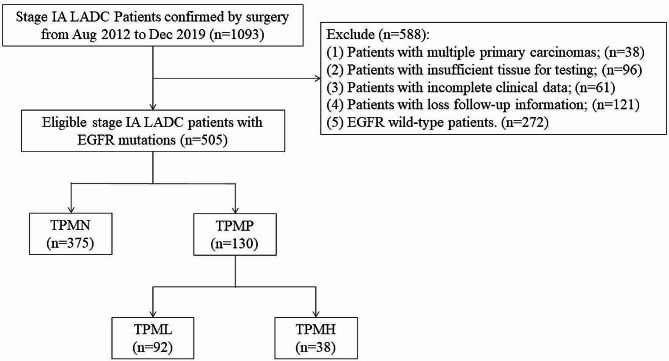
Flow diagram showing the initial study population and the numbers excluded for exclusion criteria. LADC, lung adenocarcinoma; EGFR, epidermal growth factor receptor; TPM, the proportion of micropapillary (MPP) components; TPMN, tumors without MPP subtype; TPMP, tumors with MPP subtype; TPML, tumors with 5%≤ TPM ≤ 10%; TPMH, tumors with TPM greater than 10%

Of the 1093 reviewed cases, 505 EGFR-mutated patients with pathological stage IA LADC were included in this retrospective study. The flow chart was shown in Fig. 1. There were 74.3% (375/505) in the TPMN group, 18.2% (92/505) in the TPML group, and 7.5% (38/505) in the TPMH group.

The clinical baseline characteristics of the 505 patients with the MPP subtype were compared in Table 1. The presence of MPP patterns was significantly associated with male sex (*P* = 0.044) and high pathological T stage (*P* < 0.001). Meanwhile, patients in the TPMP group were more willing to receive adjuvant therapy (*P* < 0.001), but still had a higher rate of recurrence (*P* = 0.001). A total of 45 recurrence events were recorded among the 505 patients analyzed (8.9%). In addition, 24 of those occurred in the TPMN group (6.4%), and 21 occurred in the TPMP group (16.2%).

**Table 1:** Clinical characteristics of 505 EGFR-mutated stage IA LADC patients with the MPP subtype

Variables	TPMN	TPMP	*P* value
	**(n = 375)**	**(n = 130)**	
Age			0.304
< 60	184(49.1)	57(43.8)	
≥ 60	191(50.9)	73(56.2)	
Sex			0.044
Female	231(61.6)	67(51.5)	
Male	144(38.4)	63(48.5)	
Smoking history			0.202
No	263(70.1)	81(62.3)	
Yes	60(16.0)	29(22.3)	
Unknown	52(13.9)	20(15.4)	
Tumor laterality			0.699
Left	160(42.7)	58(44.6)	
Right	215(57.3)	72(55.4)	
Resection type			0.112
Lobectomy	291(77.6)	111(85.4)	
Segmentectomy	38(10.1)	11(8.5)	
Wedge resection	46(12.3)	8(6.2)	
T stage			< 0.001
T1a	52(13.9)	4(3.1)	
T1b	219(58.4)	66(50.8)	
T1c	104(27.7)	60(46.2)	
Predominant subtype			< 0.001
Acinar	236(62.9)	87(66.9)	
Lepidic	92(24.5)	8(6.2)	
Solid	3(0.8)	3(2.3)	
Papillary	44(11.7)	27(20.8)	
Micropapillary	0(0.0)	5(3.8)	
EGFR mutation status			0.205
Others	21(5.6)	13(10.0)	
19 del	155(41.3)	54(41.5)	
L858R	199(53.1)	63(48.5)	
PAT			< 0.001
No	302(80.5)	74(56.9)	
TKI	37(9.9)	31(23.8)	
ACT	36(9.6)	25(19.2)	
Recurrence			0.001
No recurrence	351(93.6)	109(83.8)	
Recurrence	24(6.4)	21(16.2)	
Mortality			0.31
No mortality	360(96.0)	122(93.8)	
Mortality	15(4.0)	8(6.2)	

EGFR, epidermal growth factor receptor; LADC, lung adenocarcinoma; MPP, micropapillary; TPMN, tumors without MPP subtype; TPMP, tumors with MPP subtype; PAT, postoperative adjuvant treatment; TKI, epidermal growth factor receptor-tyrosine kinase inhibitor; ACT, adjuvant chemotherapy

Furthermore, we divided 130 patients into low and high percentage groups (Table 2). Patients in the TPMH group had a higher proportion of MPP patterns (*P* = 0.010) and an increased mortality risk (*P* = 0.033). However, there were no significant differences in other parameters among the TPML and TPMH groups.

**Table 2:** Clinical characteristics of 130 EGFR-mutated stage IA LADC patients with different proportions of the MPP subtype

Variables	TPML	TPMH	*P* value
	(n = 92)	(n = 38)	
Age			0.356
< 60	42(45.7)	14(36.8)	
≥ 60	50(54.3)	24(63.2)	
Sex			0.585
Female	46(50.0)	21(55.3)	
Male	46(50.0)	17(44.7)	
Smoking history			0.485
No	56(60.9)	25(65.8)	
Yes	23(25.0)	6(15.8)	
Unknown	13(14.1)	7(18.4)	
Tumor laterality			0.986
Left	41(44.6)	17(44.7)	
Right	51(55.4)	21(55.3)	
Resection type			0.662
Lobectomy	77(83.7)	34(89.5)	
Segmentectomy	9(9.8)	2(5.3)	
Wedge resection	6(6.5)	2(5.3)	
T stage			0.408
T1a	3(3.3)	1(2.6)	
T1b	50(54.3)	16(42.1)	
T1c	39(42.4)	21(55.3)	
Predominant subtype			0.01
Acinar	64(69.6)	23(60.5)	
Lepidic	5(5.4)	3(7.9)	
Solid	2(2.2)	1(2.6)	
Papillary	21(22.8)	6(15.8)	
Micropapillary	0(0.0)	5(13.2)	
EGFR mutation status			0.655
Others	8(8.7)	5(13.2)	
19 del	40(43.5)	14(36.8)	
L858R	44(47.8)	19(50.0)	
PAT			0.788
No	52(56.5)	22(57.9)	
TKI	21(22.8)	10(26.3)	
ACT	19(20.7)	6(15.8)	
Recurrence			0.329
No recurrence	79(85.9)	30(78.9)	
Recurrence	13(14.1)	8(21.1)	
Mortality			0.033
No mortality	89(96.7)	33(86.8)	
Mortality	3(3.3)	5(13.2)	

EGFR, epidermal growth factor receptor; LADC, lung adenocarcinoma; MPP, micropapillary; TPM, the proportion of MPP components; TPML, tumors with 5% ≤ TPM ≤ 10%; TPMH, tumors with TPM greater than 10%; PAT, postoperative adjuvant treatment; TKI, epidermal growth factor receptor-tyrosine kinase inhibitor; ACT, adjuvant chemotherapy

### Survival analyses

**Fig. 2:**
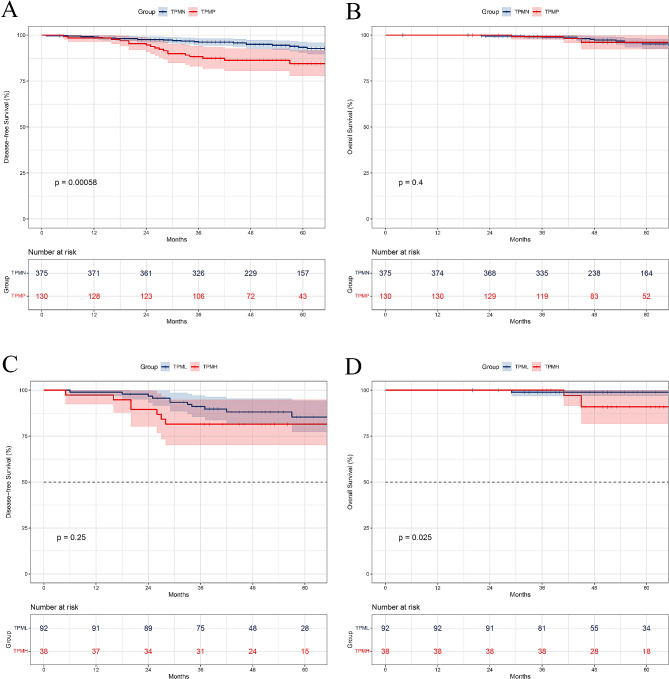
Kaplan-Meier survival curves for (**A** and **C**) disease-free and (**B** and **D**) overall survival in groups according to different proportions of MPP components. MPP, micropapillary; TPM, the proportion of MPP components; TPMN, tumors without MPP subtype; TPMP, tumors with MPP subtype; TPML, tumors with 5%≤ TPM ≤ 10%; TPMH, tumors with TPM greater than 10%

The Kaplan-Meier curves for DFS and OS in different groups are shown in Fig. 2. The median follow-up time after surgery was 57.65 months (4.0–116.0, 95% CI: 55.94–59.37). In terms of DFS, survival was significantly better in patients without the MPP pattern than in patients with ≥ 5% of the MPP pattern(TPMP vs. TPMN, 84.5% vs. 93.4%, *P* = 0.006, Fig. 2A). However, the presence of MPP was not a significant prognostic factor for OS (TPMP vs. TPMN, 95.2% vs. 96.1%, *P* = 0.400, Fig. 2B). Considering the proportion of MPP components, although there was no significant difference in DFS, the overall survival trend was worse in the high proportion group than in the low proportion group (TPMH vs. TPML, DFS: 81.6% vs. 85.4%, *P* = 0.254; OS: 91.0% vs. 98.9%, *P* = 0.025, Fig. 2C and D).

Univariable and multivariable analysis were used to explore the factors affecting DFS and OS for EGFR-mutated stage IA patients, adjusting for age, sex, smoking history, tumor laterality, resection type, T stage, TPM group, predominant subtype, and EGFR mutation status (Table 3). Notably, TPM greater than 10% was identified as an independent prognostic factor for both DFS (*P* = 0.013) and OS (*P* = 0.017). However, a low proportion of MPP components was associated only with a shortened DFS (*P* = 0.047).

**Table 3:** Univariable and multivariable Cox regressions for disease-free survival and overall survival in 505 EGFR-mutated stage IA LADC patients

Variables	N	Disease-Free Survival			Overall Survival		
		Univariable		Multivariable	Univariable		Multivariable
		HR (95%CI)	*P* value	*P* value	HR (95%CI)	*P* value	*P* value
Age							
< 60	240	Reference	0.167		Reference	0.04	0.108
≥ 60	265	1.529(0.837–2.795)			2.650(1.044–6.725)		
Sex							
Female	298	Reference	0.505		Reference	0.431	
Male	207	1.220(0.679–2.193)			0.708(0.300-1.672)		
Smoking history							
No	345	Reference	0.818		Reference	0.742	
Yes	89	1.241(0.605–2.549)	0.556		0.633(0.184–2.178)	0.469	
Unknown	71	0.953(0.395–2.299)	0.916		1.077(0.360–3.227)	0.895	
Tumor laterality							
Left	218	Reference	0.373		Reference	0.137	
Right	287	0.766(0.427–1.376)			0.535(0.234–1.221)		
Resection type							
Lobectomy	402	Reference	0.493		Reference	0.005	0.01
Segmentectomy	49	0.711(0.219–2.311)	0.571		0.570(0.075–4.302)	0.586	0.615
Wedge resection	54	1.547(0.647–3.698)	0.327		4.775(1.794–12.706)	0.002	0.004
T stage							
T1a	56	Reference	0.008	0.043	Reference	0.14	
T1b	285	1.180(0.346–4.025)	0.792	0.998	0.847(0.180–3.990)	0.834	
T1c	164	2.939(0.887–9.740)	0.078	0.22	2.018(0.454–8.969)	0.356	
TPM group							
TPMN	375	Reference	0.002	0.021	Reference	0.061	0.046
TPML	92	2.310(1.176–4.540)	0.015	0.047	0.759(0.219–2.629)	0.664	0.903
TPMH	38	3.644(1.635–8.117)	0.002	0.013	3.101(1.125–8.544)	0.029	0.017
Predominant subtype							
Acinar	323	Reference	0.578		Reference	0.699	
Lepidic	100	0.644(0.267–1.554)	0.327		0.346(0.080–1.497)	0.156	
Solid	6	0	0.975		0	0.989	
Papillary	71	1.388(0.657–2.934)	0.391		0.689(0.202–2.342)	0.55	
Micropapillary	5	2.432(0.331–17.893)	0.383		0	0.987	
EGFR mutation status							
Others	34	Reference	0.486		Reference	0.317	
19 del	209	0.657(0.223–1.931)	0.445		0.562(0.124–2.545)	0.455	
L858R	262	0.533(0.182–1.563)	0.252		0.347(0.074–1.626)	0.179	

EGFR, epidermal growth factor receptor; LADC, lung adenocarcinoma; HR, hazard ratio; CI, confidence interval; TPM, the proportion of micropapillary components; TPMN, tumors without micropapillary subtype; TPML, tumors with 5%≤ TPM ≤ 10%; TPMH, tumors with TPM greater than 10%

### Treatment efficacy

**Fig. 3:**
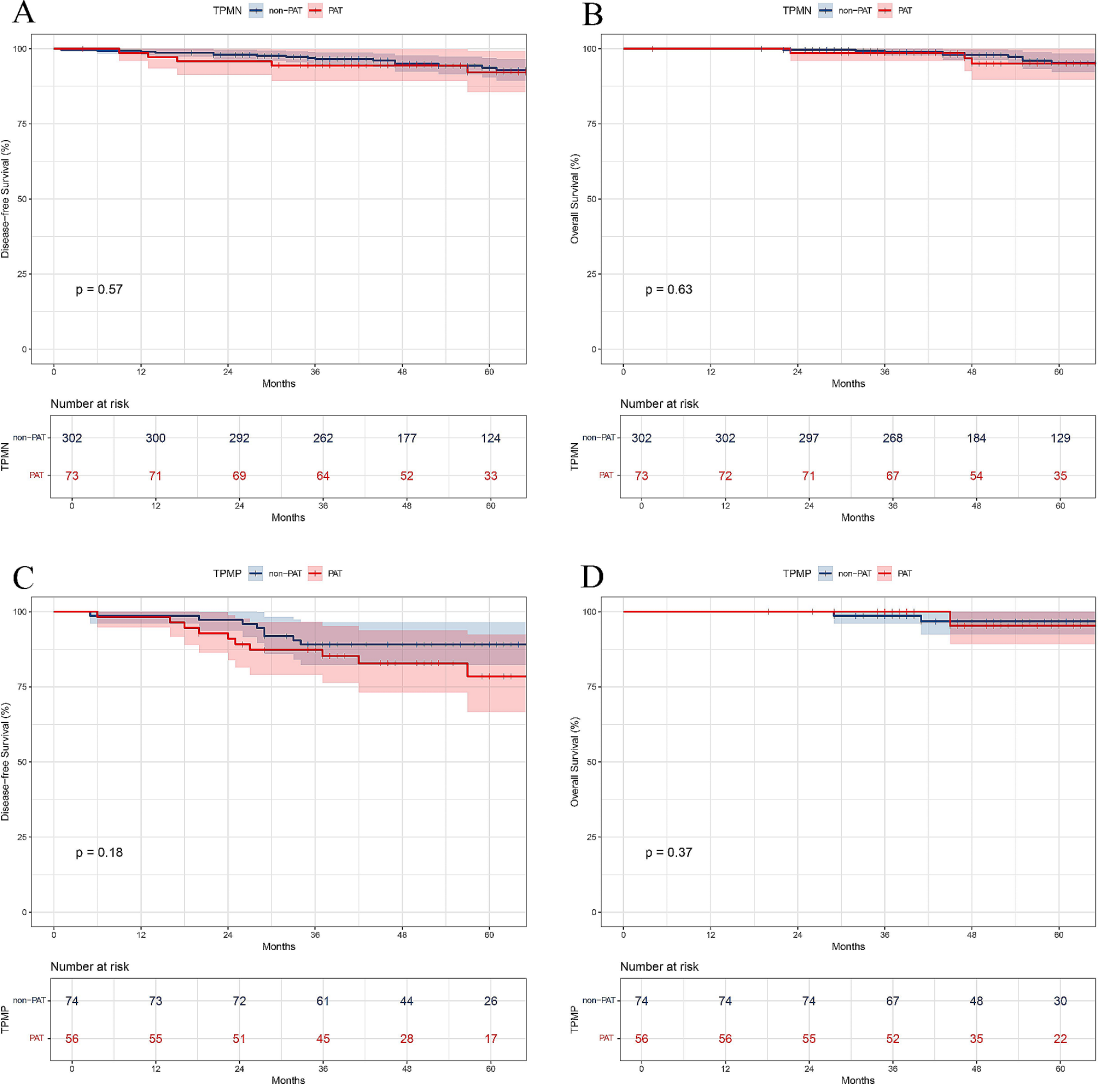
Kaplan-Meier survival curves for (**A** and **C**) disease-free and (**B** and **D**) overall survival according to the PAT in the TPMN (**A** and **B**) and TPMP (**C** and **D**) groups. PAT, postoperative adjuvant treatment; non-PAT, observation; TPMN, tumors without micropapillary (MPP) subtype; TPMP, tumors with MPP subtype

Among the 505 patients, 129(25.5%) who received adjuvant treatment tended to have a higher T stage (*P* < 0.001) and a larger amount of the MPP component (*P* < 0.001, Table 4). Patients without MPP patterns did not benefit from PAT (non-PAT group vs. PAT group, 5-year DFS, 96.6% vs. 88.0%, *P* = 0.574; 5-year OS, 98.9% vs. 90.9%; *P* = 0.632; Fig. 3A and B), regardless of observing EGFR-TKIs (non-PAT group vs. TKI group, 5-year DFS, 96.6% vs. 83.9%, *P* = 0.663; 5-year OS, 98.9% vs. 87.8%, *P* = 0.675; Supplementary Fig. 1) or chemotherapy (non-PAT group vs. ACT group, 5-year DFS, 96.6% vs. 91.4%,*P* = 0.674; 5-year OS, 98.9% vs. 94.1%, *P* = 0.764; Supplementary Fig. 1).

**Table 4:** Clinical characteristics of 505 EGFR-mutated stage IA LADC patients with different treatments

Variables	Surgical Intervention Alone	PAT	*P* value
	(n = 376)	(n = 129)	
Age			0.637
< 60	181(48.1)	59(45.7)	
≥ 60	195(51.9)	70(54.3)	
Sex			0.517
Female	225(59.8)	73(56.6)	
Male	151(40.2)	56(43.4)	
Smoking history			0.21
No	263(69.9)	82(63.6)	
Yes	66(17.6)	23(17.8)	
Unknown	47(12.5)	24(18.6)	
Tumor laterality			0.58
Left	165(43.9)	53(41.1)	
Right	211(56.1)	76(58.9)	
Resection type			0.823
Lobectomy	299(79.5)	103(79.8)	
Segmentectomy	38(10.1)	11(8.5)	
Wedge resection	39(10.4)	15(11.6)	
T stage			< 0.001
T1a	49(13.0)	7(5.4)	
T1b	223(59.3)	62(48.1)	
T1c	104(27.7)	60(46.5)	
TPM group			< 0.001
TPMN	302(80.3)	73(56.6)	
TPML	52(13.8)	40(31.0)	
TPMH	22(5.9)	16(12.4)	
Predominant subtype			0.003
Acinar	240(63.8)	83(64.3)	
Lepidic	84(22.3)	16(12.4)	
Solid	2(0.5)	4(3.1)	
Papillary	45(12.0)	26(20.2)	
Micropapillary	5(1.3)	0(0.0)	
EGFR mutation status			0.109
Others	21(5.6)	13(10.1)	
19 del	163(43.4)	46(35.7)	
L858R	192(51.1)	70(54.3)	

EGFR, epidermal growth factor receptor; LADC, lung adenocarcinoma; PAT, postoperative adjuvant treatment; TPM, the proportion of micropapillary components; TPMN, tumors without micropapillary subtype; TPML, tumors with 5%≤ TPM ≤ 10%; TPMH, tumors with TPM greater than 10%

Subsequently, we evaluated the postoperative treatment outcomes for patients with the MPP component exceeding 5%. For stage IA patients in the TPMP group, the differences in DFS and OS between those receiving PAT and those who did not were not statistically significant (non-PAT group vs. PAT group, 5-year DFS, 89.1% vs. 78.5%, *P* = 0.176; 5-year OS, 96.8% vs. 95.3%, *P* = 0.368; Fig. 3C and D), whether they received EGFR-TKIs (non-PAT group vs. TKI group, 5-year DFS, 89.1% vs. 86.4%, *P* = 0.864; 5-year OS, 96.8% vs. 95.2%, *P* = 0.642; Supplementary Fig. 2) or chemotherapy (non-PAT group vs. ACT group, 5-year DFS, 89.1% vs. 71.0%, *P* = 0.234; 5-year OS, 96.8% vs. 95.5%, *P* = 0.643; Supplementary Fig. 2).

Kaplan–Meier curves further revealed that patients with different proportions of the MPP component had comparable prognoses whether they received PAT therapy or not. (non-PAT group vs. PAT group, TPML: 5-year DFS, 92.1% vs. 77.1%, *P* = 0.138; 5-year OS, 98.1% vs. 100.0%, *P* = 0.451; TPMH: 5-year DFS, 81.8% vs. 81.3%, *P* = 0.956; 5-year OS, 95.2% vs. 84.6%, *P* = 0.407; Supplementary Fig. 3).

## Discussion

This retrospective study reports real-world data for the clinicopathological characteristics and survival outcomes of EGFR-mutated stage IA LADC patients. Importantly, it demonstrates that the high proportions of the MPP subtype significantly influence prognostic outcomes. Since a new histological classification of LADC was proposed by IASLC/ATS/ERS, the impact of the MPP component on the survival of early lung cancer had attracted more attention. Previous research has proven that early-stage lung tumors with the MPP subtype show a higher more frequency of lympho-vascular invasion (LVI) and spread through air spaces (STAS) [10–13]. But there are few reports emphasizing the prognostic value of the percentages of MPP components. Qian et al. [14] conducted an analysis of stage IB LADC patients and found that the survival of the SMPP (solid/micropapillary-predominant) group was even poorer than that of the SMPM (solid/micropapillary-minor) group. As a result, according to TPM, we divided patients into three different subgroups, including TPMN (tumors without MPP subtype), TPML (tumors with 5% ≤ TPM ≤ 10%), and TPMH (TPM greater than 10%). In our study, the survival analyses have demonstrated that DFS was significantly poorer in patients with the presence of an MPP pattern (*P* = 0.006), and the OS trend was worse in the high proportion of MPP subtype group (*P* = 0.025). Meanwhile, multivariable analyses identified TPM greater than 10% as an independent prognostic factor for both DFS (*P* = 0.013) and OS (*P* = 0.017).

In the entire study cohort, the role of targeted therapy failed to be detected. Previous studies concluded the prognostic implication of EGFR mutations in resected NSCLC cases [15–19]. Ito et al. [20] demonstrated that the ratio of EGFR mutations and the risk of recurrence vary among histological subtypes in pN0M0 LADC. Exploring the clinicopathological characteristics of EGFR mutations is essential for lung cancer treatment. It is well known that MPP components of 5% or higher are a potential pathological marker for poor prognosis [8, 9, 21, 22]. Additionally, some studies have found that the frequency of MPP is higher in EGFR mutations [23–25]. Although biologically aggressive, OS is generally prolonged after receiving EGFR-TKIs [12, 13, 26]. However, the efficacy of TKIs is inconsistent among different patients, which may result from histologic features. In summary, histological subtypes of LADC with EGFR mutations can help predict the prognostic impact and therapeutic effect of TKIs. Although the National Comprehensive Cancer Network (NCCN) guideline suggest that PAT may be considered in stage IB patients with high-risk factors [27], the rationality of PAT in stage IA patients with MPP components remains controversial. Wang et al. [28] analyzed 152 stage IA LADC patients with MPP-predominant disease and revealed a better OS benefit of chemotherapy in subgroups stratified according to EGFR mutation status. However, the data excluded patients receiving targeted therapy. Yucheng et al. [29] reported that no significant difference was observed in MPP pattern stage IA patients who received postoperative chemotherapy. Regarding treatment efficacy, our study found that patients with MPP patterns did not benefit from PAT, regardless of whether they received EGFR-TKIs or chemotherapy. Kaplan-Meier curves further confirmed that patients with different proportions of MPP components had comparable prognoses whether they received PAT or not.

These findings suggest that current PAT strategies may not be sufficient to improve outcomes in EGFR-mutated stage IA LADC patients with MPP patterns. The lack of significant benefit from EGFR-TKIs and chemotherapy in this subgroup indicates the need for alternative therapeutic approaches or the identification of novel biomarkers to better stratify patients who might benefit from specific treatments. Therefore, we need closer surveillance for stage IA patients in this high-risk subgroup. Meanwhile, whether EGFR-mutated stage IA patients with MPP components can obtain survival benefits from targeted therapy or even chemotherapy needs further research based on a large sample size in the future.

There were some limitations to this study. First, it was a single-institution retrospective study, and incomplete data were inevitable. Additionally, the regimen selection and postoperative adjuvant decision were based on physician preference rather than randomization. Therefore, prospective multicenter clinical trials are warranted to validate the results in the future.

## Conclusion

The proportion of MPP components is a significant marker of poor prognosis in EGFR-mutated patients with stage IA LADC. In these patients, future clinical trials are warranted to evaluate the role of EGFR-TKIs and adjuvant chemotherapy.

## Electronic supplementary material

Below is the link to the electronic supplementary material.

**Supplementary material 1**: **Supplementary figure 1.** Kaplan-Meier survival curves for (A) disease-free and (B) overall survival according to the types of PAT in the TPMN group. TPMN, tumors without micropapillary subtype; PAT, postoperative adjuvant treatment; non-PAT, observation; TKI, epidermal growth factor receptor-tyrosine kinase inhibitor; ACT, adjuvant chemotherapy

**Supplementary material 2**: **Supplementary figure 2.** Kaplan-Meier survival curves for (A) disease-free and (B) overall survival according to the types of PAT in the TPMP group. TPMP, tumors with micropapillary subtype; PAT, postoperative adjuvant treatment; non-PAT, observation; TKI, epidermal growth factor receptor-tyrosine kinase inhibitor; ACT, adjuvant chemotherapy

**Supplementary material 3**: **Supplementary figure 3.** Kaplan-Meier survival curves for (A and C) disease-free and (B and D) overall survival according to the PAT in the TPML (A and B) and TPMH (C and D) groups. TPML, tumors with 5%≤ the proportion of MPP components (TPM) ≤ 10%; TPMH, tumors with TPM greater than 10%; PAT, postoperative adjuvant treatment; non-PAT, observation

## Data Availability

No datasets were generated or analysed during the current study.
