# Supplementary figures and images for: The impact of postoperative adjuvant therapy on EGFR-mutated stage IA lung adenocarcinoma with micropapillary pathological subtypes
Source: World J Surg Oncol. 2024 Sep 5;22:235. doi: 10.1186/s12957-024-03429-y (PMC11375949; doi:10.1186/s12957-024-03429-y)

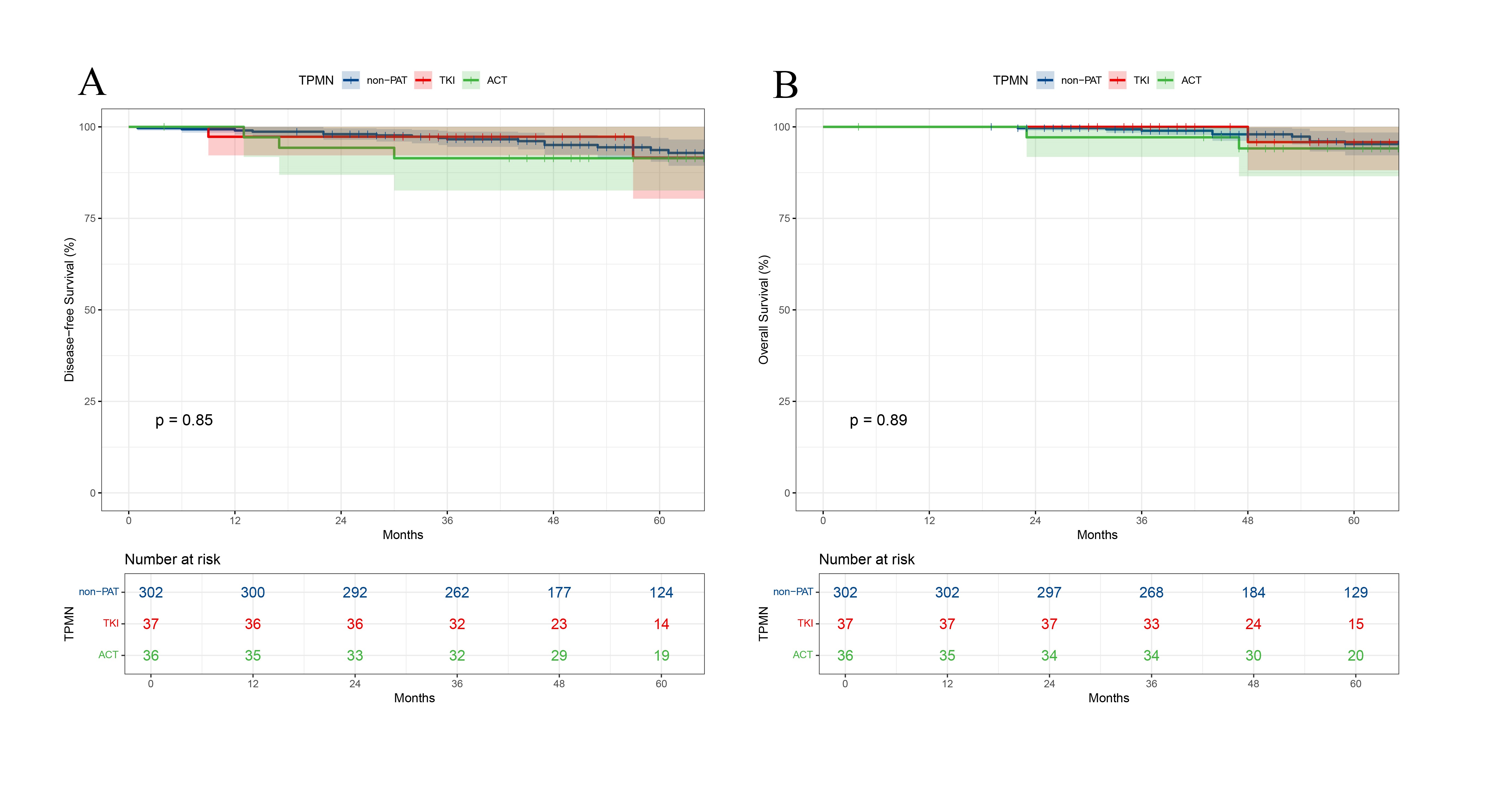

Supplement: Supplementary file 1 — Supplementary material 1: Supplementary figure 1. Kaplan-Meier survival curves for (A) disease-free and (B) overall survival according to the types of PAT in the TPMN group. TPMN, tumors without micropapillary subtype; PAT, postoperative adjuvant treatment; non-PAT, observation; TKI, epidermal growth factor receptor-tyrosine kinase inhibitor; ACT, adjuvant chemotherapy [file 12957_2024_3429_MOESM1_ESM.jpg]

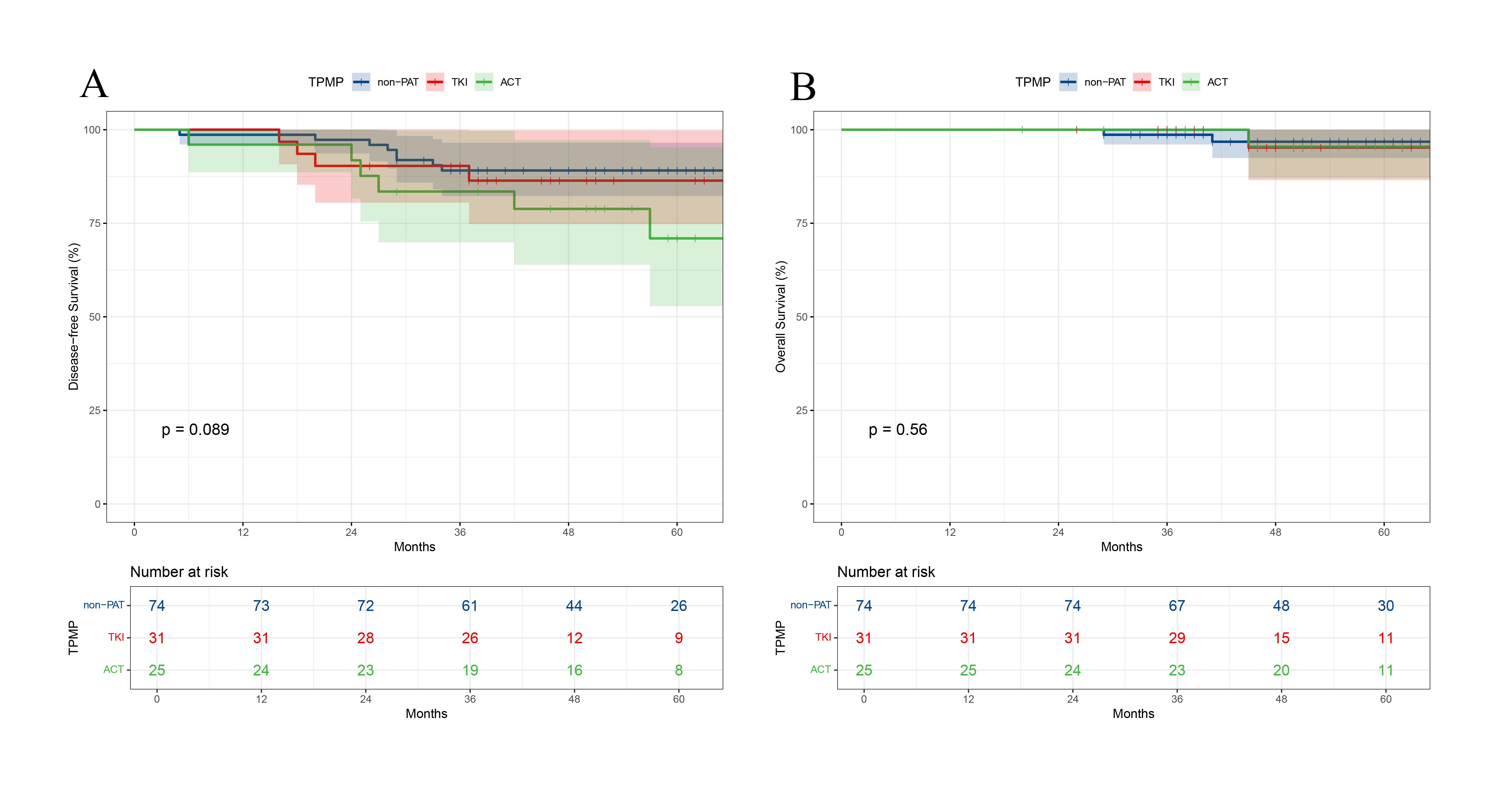

Supplement: Supplementary file 2 — Supplementary material 2: Supplementary figure 2. Kaplan-Meier survival curves for (A) disease-free and (B) overall survival according to the types of PAT in the TPMP group. TPMP, tumors with micropapillary subtype; PAT, postoperative adjuvant treatment; non-PAT, observation; TKI, epidermal growth factor receptor-tyrosine kinase inhibitor; ACT, adjuvant chemotherapy [file 12957_2024_3429_MOESM2_ESM.jpg]

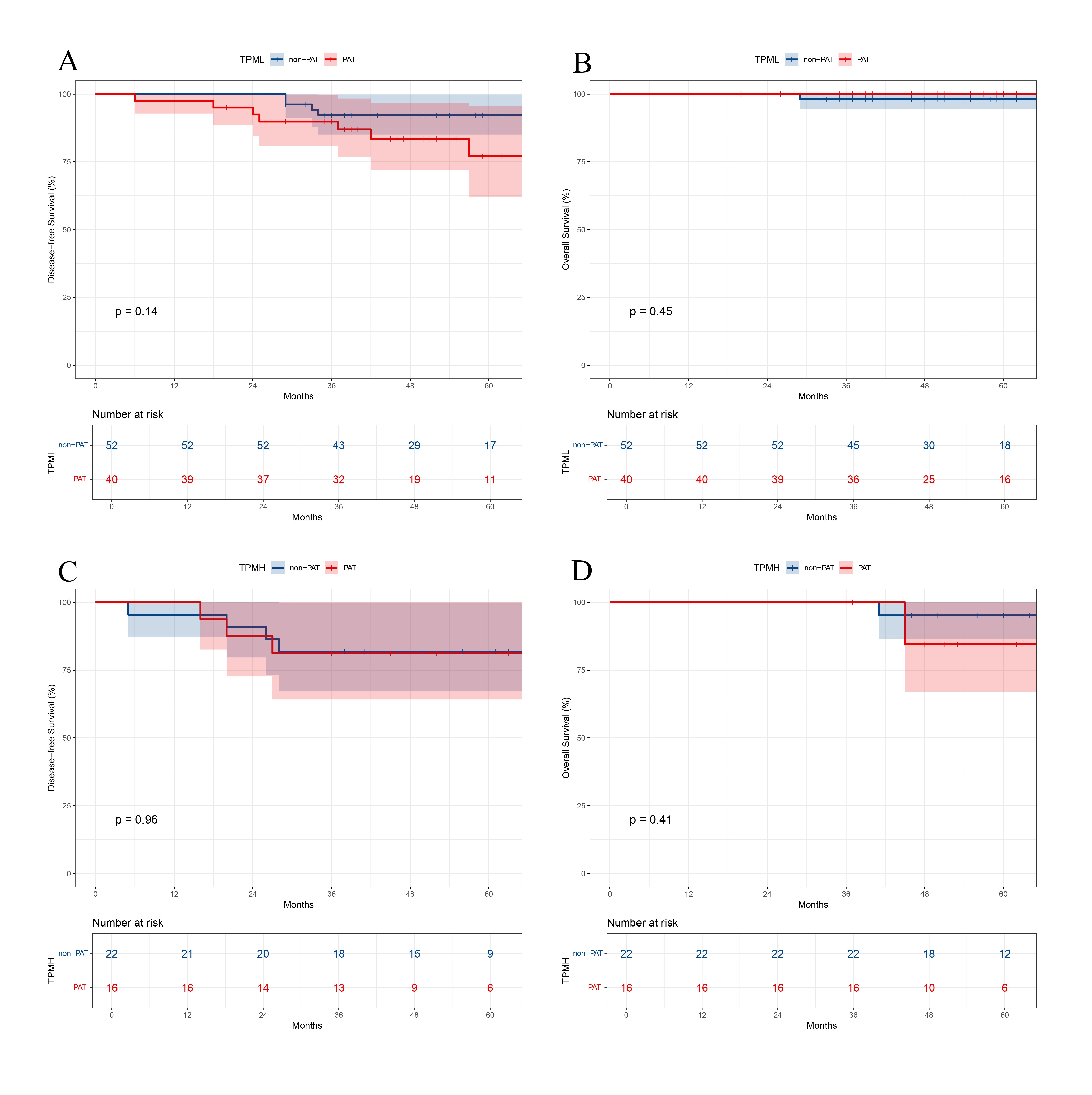

Supplement: Supplementary file 3 — Supplementary material 3: Supplementary figure 3. Kaplan-Meier survival curves for (A and C) disease-free and (B and D) overall survival according to the PAT in the TPML (A and B) and TPMH (C and D) groups. TPML, tumors with 5%≤ the proportion of MPP components (TPM) ≤ 10%; TPMH, tumors with TPM greater than 10%; PAT, postoperative adjuvant treatment; non-PAT, observation [file 12957_2024_3429_MOESM3_ESM.jpg]
